# Supplementary material for: Comparative Analysis of Phytochemical Profiles and Antioxidant Activities between Sweet and Sour Wampee (Clausena lansium) Fruits
Source: Foods. 2022 Apr 25;11(9):1230. doi: 10.3390/foods11091230 (PMC9103836; doi:10.3390/foods11091230)
Supplement: Supplementary file 1 [file foods-11-01230-s001.zip › foods-1672852-supplementary.pdf]

THP

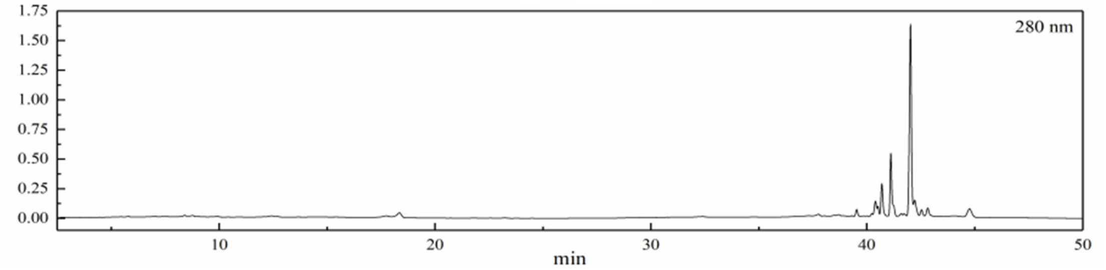

ZFHP

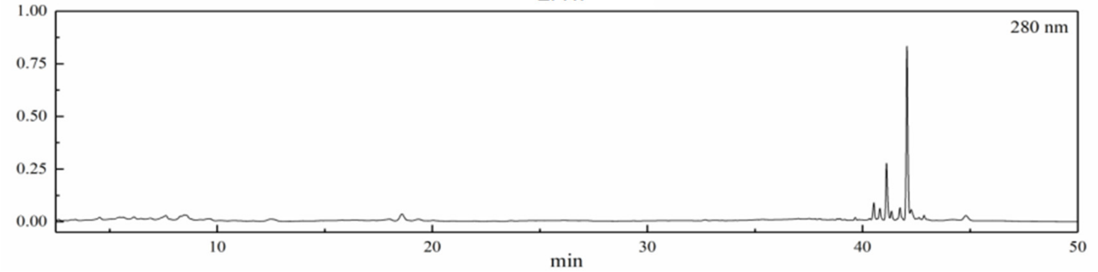

CCTP

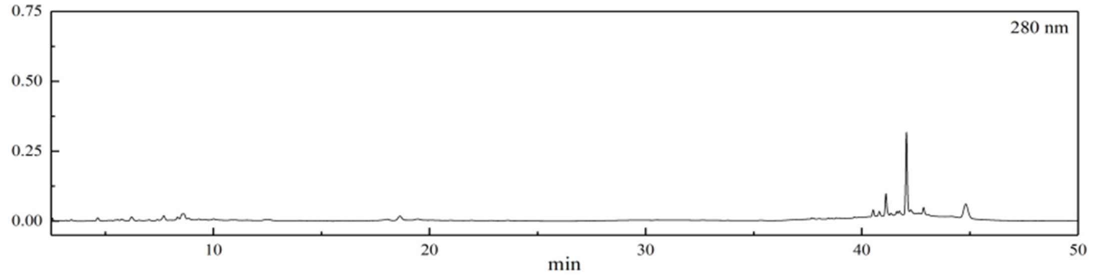

LTDH

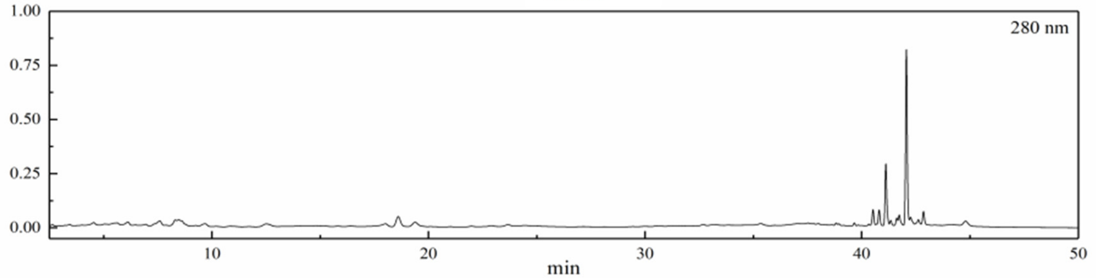

TXTP

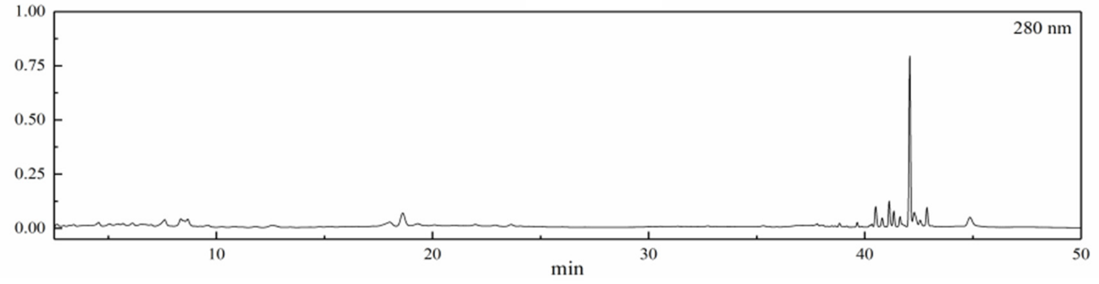

M3H

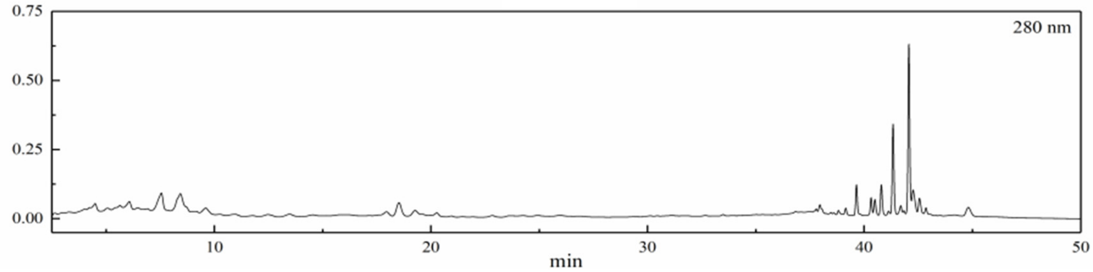

JZP

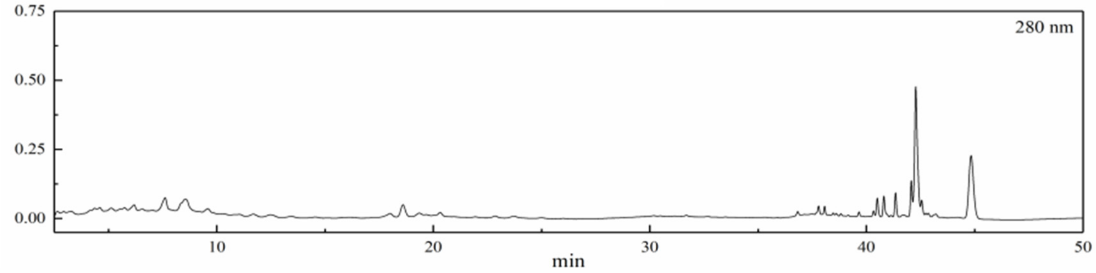

M4H

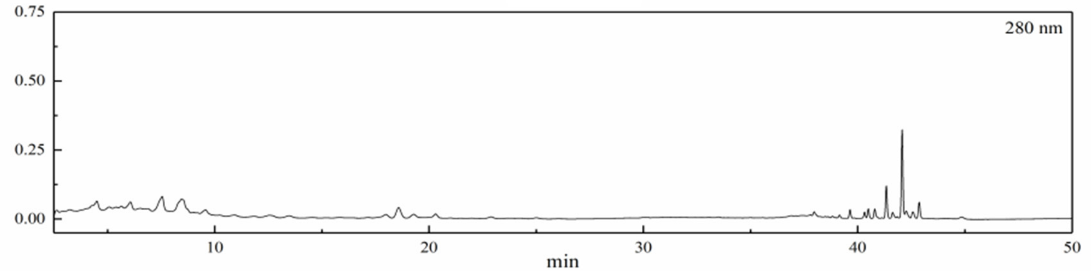

TXSP

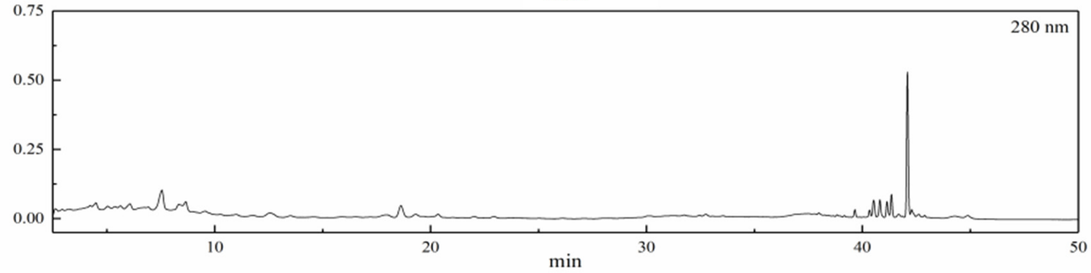

JXHP

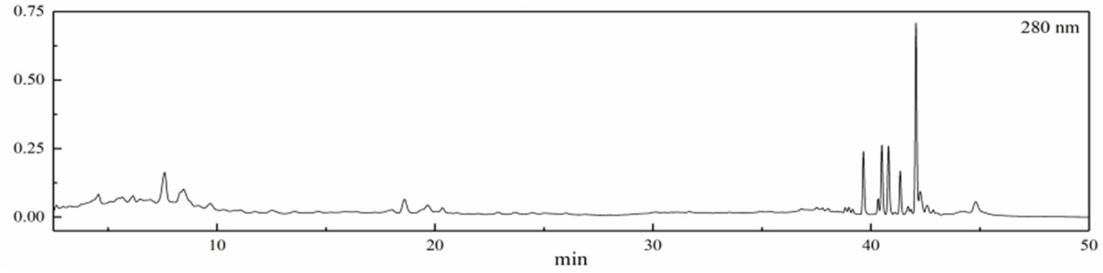

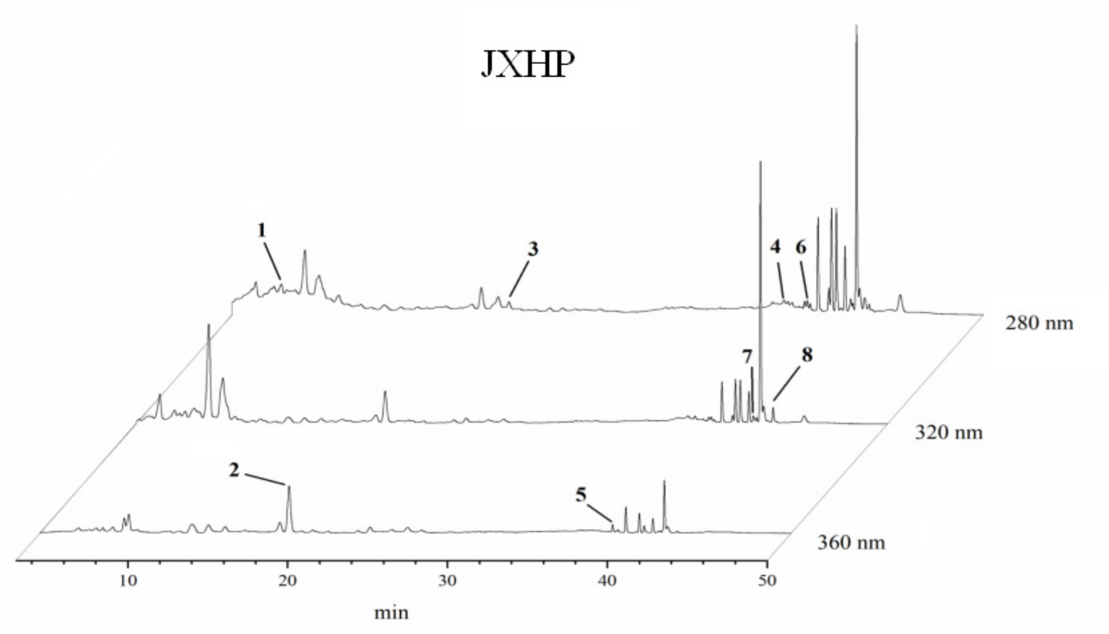

No.	Phytochemical names	Peak time(min)	Wave length(nm)
**1**	**Syringin**	**6.480**	**280**
**2**	**Rutin**	**18.515**	**360**
**3**	**Benoic acid**	**20.993**	**280**
**4**	**2-Methoxycinnamic acid**	**37.520**	**280**
**5**	**Kaempferol**	**38.710**	**360**
**6**	**Hesperetin**	**38.967**	**280**
**7**	**Nobiletin**	**41.604**	**320**
**8**	**Tangeretin**	**42.602**	**320**
